# Supplementary material for: In Silico Study Identified Methotrexate Analog as Potential Inhibitor of Drug Resistant Human Dihydrofolate Reductase for Cancer Therapeutics
Source: Molecules. 2020 Jul 31;25(15):3510. doi: 10.3390/molecules25153510 (PMC7435474; doi:10.3390/molecules25153510)
Supplement: Supplementary file 1 [file molecules-25-03510-s001.pdf]

Table S1. List of analog compounds of methotrexate downloaded from ZINC[15] with the description their ZINC ids, molecular formulas and 2D structures representations.

Compound No.	2D Structure	Molecular Formula	ZINC ID
**Compound 1**	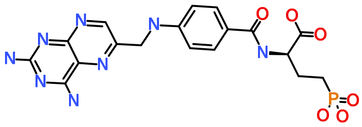	C18 H21 N8 O6 P	ZINC000028764059
**Compound 2**	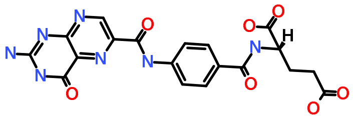	C19 H17 N7 O7	ZINC000068590381
**Compound 3**	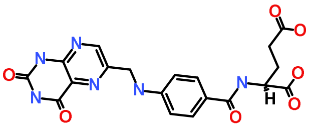	C19 H18 N6 O7	ZINC000001698171
**Compound 4**	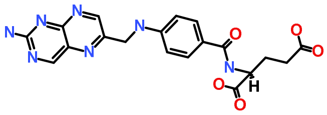	C19 H19 N7 O5	ZINC000002944400
**Compound 5**	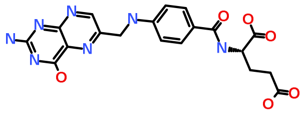	C19 H19 N7 O6	ZINC000008577218
**Compound 6**	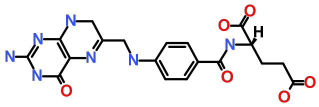	C19 H21 N7 O6	ZINC000004228265
**Compound 7**	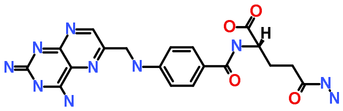	C19 H22 N10 O4	ZINC000504557614
**Compound 8**	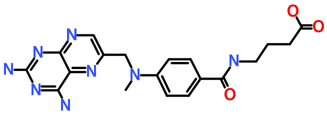	C19 H22 N8 O3	ZINC000001653972
**Compound 9**	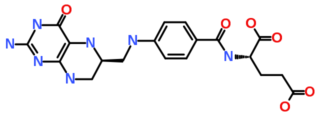	C19 H23 N7 O6	ZINC000004228237
**Compound 10**	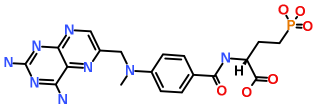	C19 H23 N8 O6 P	ZINC000028763233
**Compound 11**	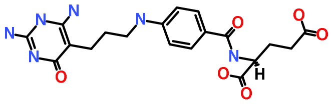	C19 H24 N6 O6	ZINC000008642279

**Compound 12**	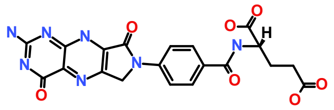	C20 H17 N7 O7	ZINC000002035058
**Compound 13**	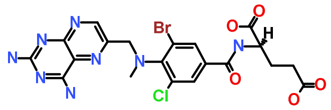	C20 H20 Br Cl N8 O5	ZINC000008619351
**Compound 14**	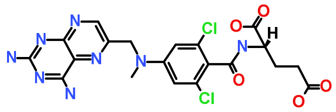	C20 H20 Cl2 N8 O5	ZINC000001728420
**compound15**	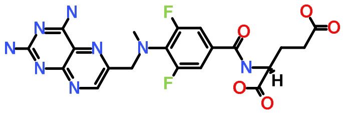	C20 H20 F2 N8 O5	ZINC000001722686
**Compound 16**	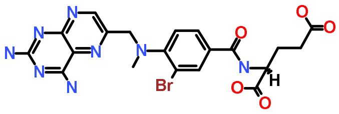	C20 H21 Br N8 O5	ZINC000001643916
**Compound 17**	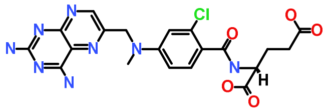	C20 H21 Cl N8 O5	ZINC000008628726
**Compound 18**	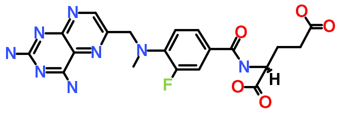	C20 H21 F N8 O5	ZINC000001698176
**Compound 19**	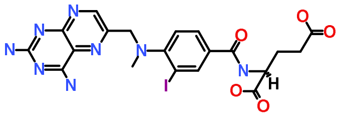	C20 H21 I N8 O5	ZINC000001722685
**Compound 20**	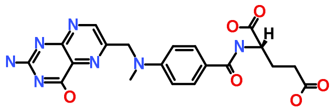	C20 H21 N7 O6	ZINC000008214609
**Compound 21**	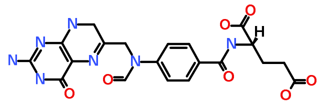	C20 H21 N7 O7	ZINC000011536135
**Compound 22**	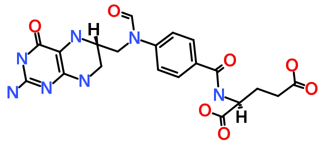	C20 H22 N7 O6	ZINC000004228269
**Compound 23**	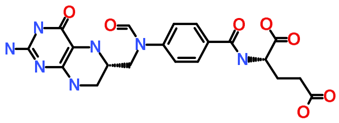	C20 H23 N7 O7	ZINC000004228247

**Compound 24**	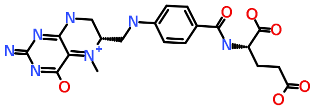	C20 H24 N7 O6	ZINC000070451053
**Compound 25**	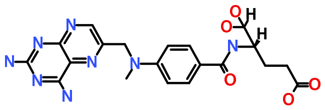	C20 H24 N8 O5	ZINC000584577528
**Compound 26**	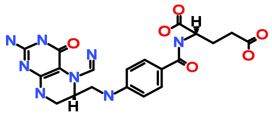	C20 H24 N8 O6	ZINC000004228294
**Compound 27**	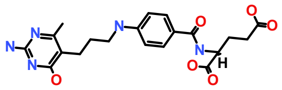	C20 H25 N5 O6	ZINC000008626527
**Compound 28**	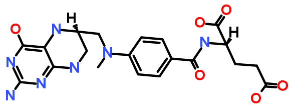	C20 H25 N7 O6	ZINC000013508844
**Compound 29**	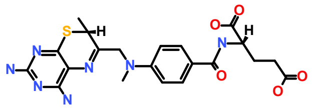	C21 H25 N7 O5 S	ZINC000033884989
**Compound 30**	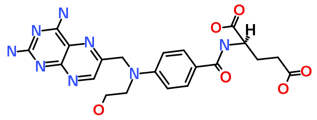	C21 H24 N8 O6	ZINC000013508844
**Compound 31**	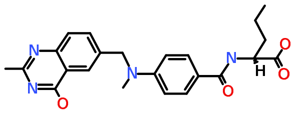	C23 H26 N4 O4	ZINC000725274512
**Compound 32**	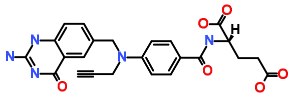	C24 H23 N5 O6	ZINC000008655373

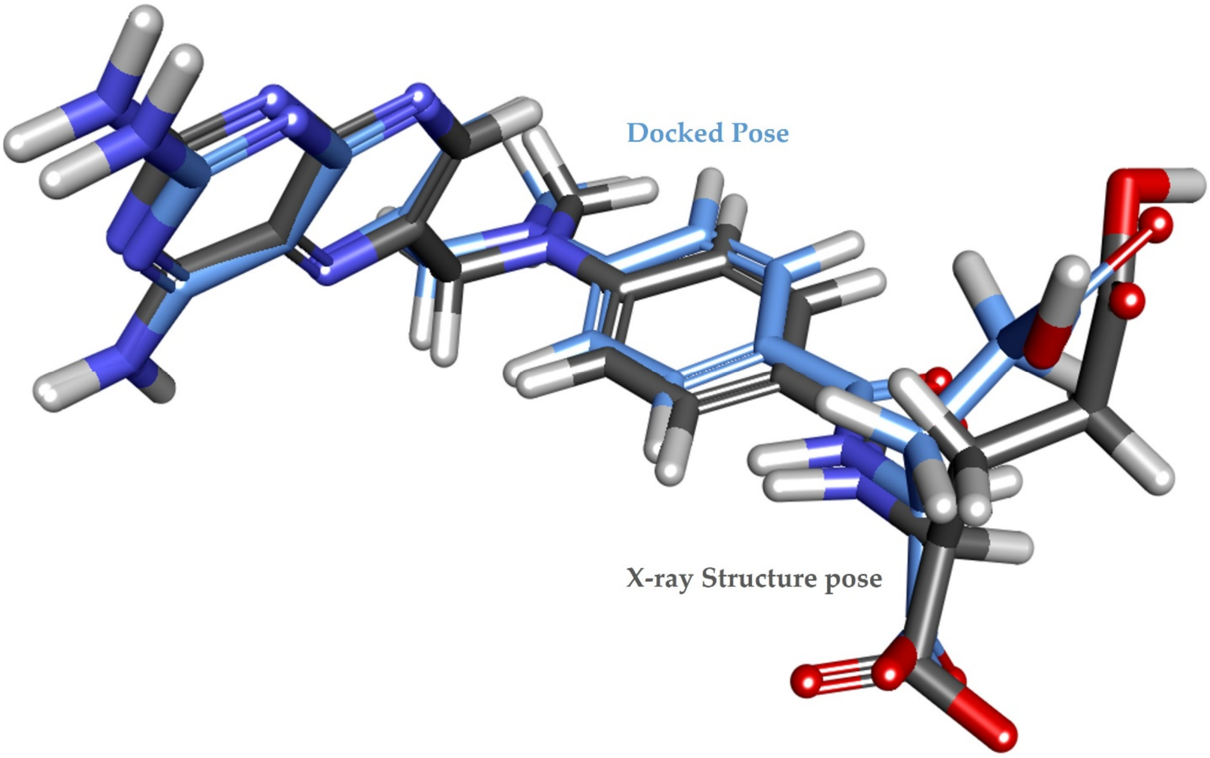

Figure S1. Superimposition of docked pose and crystal structure pose of methotrexate in wild type hDHFR. A low RMSD difference of 0.58Å suggested high suitability of docking protocol.
